# An Advanced Characterization Method for the Elastic Modulus of Nanoscale Thin-Films Using a High-Frequency Micromechanical Resonator

**DOI:** 10.3390/ma10070806

**Published:** 2017-07-15

**Authors:** Yun Young Kim

**Affiliations:** Division of Mechanical, Automotive, and Robot Component Engineering, Dong-eui University, Busan 47340, Korea; ykim@deu.ac.kr; Tel.: +82-51-890-1649

**Keywords:** micromechanical resonator, materials characterization, thin-film, elastic modulus

## Abstract

Nanoscale materials have properties that frequently differ from those of their bulk form due to the scale effect, and therefore a measurement technique that can take account of such material characteristics with high accuracy and sensitivity is required. In the present study, advanced nanomechanical metrology was developed for evaluation of elastic properties of thin-film materials. A 52 nm thick chromium (Cr) film was deposited on a high-speed micromechanical resonator using an e-beam evaporator, and the structure was excited to resonate using an ultrasonic platform. The resonant frequencies for the first and second flexural vibration modes were measured using laser interferometry, and they were compared to analytical estimation from the classical beam theory. Results show that the experimental data are in excellent agreement with the theory, within 1% of the relative error, and a mass sensitivity up to 10.5 Hz/fg was achieved. Thus, the scale effect that reduced the Young’s modulus of Cr by 49.8% compared to its bulk property was correctly recognized by the proposed method.

## 1. Introduction

Mechanical properties of nanoscale thin-films have drawn significant attention in the fields of science and engineering, not only for intellectual reasons but for practical applications in microelectromechanical systems (MEMS) and semiconductor devices. For example, the state-of-the-art three-dimensional memory devices require micro/nanomechanical stability design and simulation in order to prevent structural failures during the microfabrication process, unlike their predecessors based on planar device technologies [1]. During the design, however, properties of bulk materials cannot be used because they frequently deviate from literature values due to the scale effect and also vary depending on process conditions. Therefore, it is essential to develop an evaluation technique that accounts for such material characteristics.

Among many techniques such as acoustic microscopy [2], nanoindentation testing [3], microtensile testing [4], laser ultrasonics [5], and so on, micromechanical resonator-based sensing provides an effective and convenient way to study characteristics of thin-films and nanoscale materials [6]. In the microresonator testing, attachment of molecules onto the surface of a cantilever beam-like MEMS structure results in a shift of its resonant frequency. Therefore, properties of the attached materials can be calculated once the frequency shift is measured and theoretically analyzed. To improve the microresonator’s performance, the following measures have been taken: increasing the operating frequency by using higher order resonance modes and decreasing the resonator mass by reducing the volume of the structure, due to the mass sensitivity (*S*) of the microresonator being equal to the resonant frequency shift (Δ*f*) divided by the mass addition (Δ*m*) [7]:

(1) S=Δf/Δm

Reducing the volume of the structure, however, addresses problems in microfabrication and instrumentation [8]. For example, a position sensitive detector (PSD)-equipped atomic force microscopy (AFM)-based approach is conventionally used for the evaluation, but its sensitivity is limited by instrumentation issues arising from the minimum angle of beam deflection or, in turn, the minimum length of the micromechanical resonator, since it depends on the optical lever method for detection. Increasing the resonant frequency is also limited by the bandwidth of PSD. Although certain approaches have been demonstrated to increase the operation frequency to the megahertz (MHz) and gigahertz range [9,10,11,12], a typical AFM probe still resonates in the lower kilohertz frequency range. Therefore, faster actuation and sensing are required to enhance the resolution.

Here, an advanced nanomaterials characterization method is introduced using a micromechanical resonator actuating in the MHz frequency range. The resonant frequency shift of the resonator upon deposition of a nanoscale thin-film, which is a chromium (Cr) layer for demonstration in this study, is detected so that the film’s elastic modulus can be evaluated. The time-domain waveform of the resonance signal is measured using a path-stabilized Michelson interferometer—instead of using the optical lever method—for the high frequency detection, and the vibration mode of the micromechanical resonator is identified using the Euler–Bernoulli beam theory. The advantage of the proposed technique is that increasing the actuation and detection frequencies to the MHz range makes it possible to evaluate nanomaterials with high sensitivity and accuracy.

## 2. Materials and Methods

### 2.1. Materials

**Figure 1:**
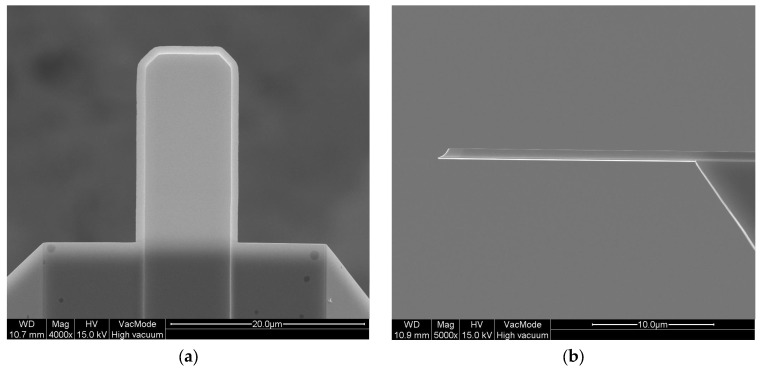
Field emission scanning electron microscopy pictures of the micromechanical resonator: (**a**) top-view; (**b**) side-view.

A high-speed micromechanical resonator (SD-USC-F1.2-k7.3-TL, Nanoworld AG, Neuchâtel, Switzerland) was used in this study, as shown in Figure 1. The length (*L*) and width (*b*) of the structure are 20 μm and 10 μm, respectively. It has 30 nm thick gold (Au) layers on the top and bottom sides of the 670 nm thick quartz core, for optical reflection of a detection laser beam.

**Figure 2:**
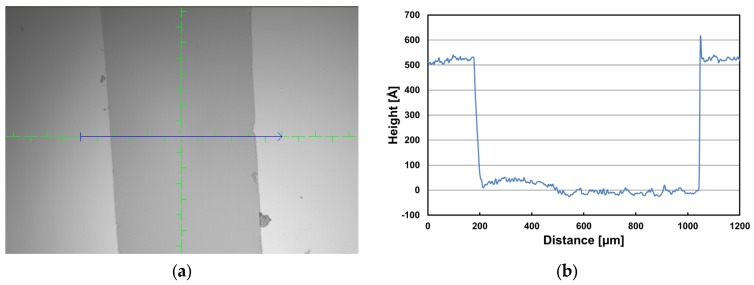
(**a**) A microscopy image of the chromium-coated silicon wafer. The dark area in the center was partially covered during the film deposition for the thickness measurement along the blue arrow; (**b**) thickness measurement data using a profilometer.

A 52 nm thick Cr layer was additionally deposited on the top surface using an e-beam evaporator (FC-2000, Temescal Systems, Livermore, CA, USA). The chamber pressure and deposition rate were maintained at 1.1 × 10^−7^ Torr and 2.0 Å/s, respectively, during the process. The film thickness was checked using a profilometer (P6, KLA Tencor, Milpitas, CA, USA) after the deposition was complete—since the film thickness is an important parameter that influences the Young’s modulus of Cr film in this experiment, a bare silicon (Si) (100) wafer with a polished surface was also installed in the chamber and partially covered to measure the thickness of the Cr layer. The measurement data in Figure 2 show that the film thickness is approximately 52 ± 2 nm.

**Figure 3:**
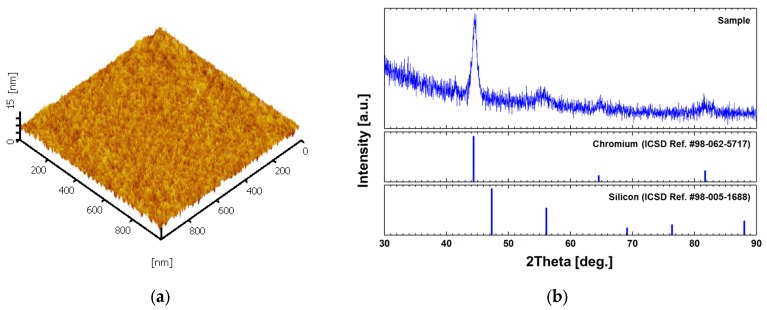
Sample characteristics. (**a**) An atomic force microscopy image of the chromium film on the micromechanical resonator. The surface roughness (*R_a_*) is 0.79 nm; (**b**) grazing incidence X-ray diffraction (GIXRD) peaks on the chromium film on a Si(100) substrate.

To study microstructures of the film, the surface morphology was imaged using an atomic force microscopy (AFM L-trace II, Hitachi High-Tech Science Corporation, Tokyo, Japan) operating in a tapping mode. 512 × 512 pixels were measured within an area of 1 × 1 μm^2^ at a scanning speed of 1 Hz. In addition, grazing incidence X-ray diffraction (GIXRD) patterns were measured (X’pert Pro, PANalytical B.V., Almelo, Netherlands) in the range of 30°–90° with an angular step of 0.02 at a scan step time of 1 s. Due to the difficulty of directly measuring the chromium film on the microresonator surface with an area of only 20 × 10 μm^2^, the reference sample (the same film on a 4 inch Si(100) wafer) was measured instead. The AFM image introduced in Figure 3a reveals that the surface roughness (*R_a_*) is 0.79 nm. Figure 3b shows that the GIXRD patterns and peaks are located at 2θ = 44°, 65°, and 82°, corresponding to the Cr(110), Cr(200), and Cr(211) planes, respectively, according to the Inorganic Crystal Structure Database (ICSD), reference code No. 98-062-5717. The stick patterns for silicon (ICSD reference code No. 98-005-1688) was also plotted together in Figure 3b.

### 2.2. Characterization Methods

**Figure 4:**
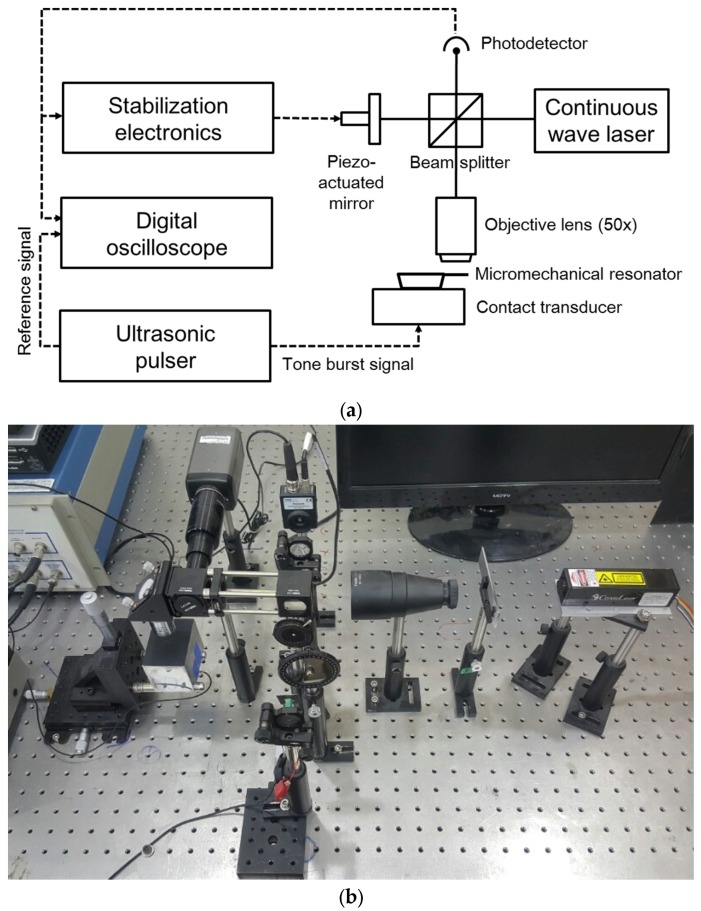
(**a**) A schematic representation of the experimental apparatus; (**b**) a picture of the actual interferometer setup.

Figure 4a shows a schematic of the test apparatus for the resonant frequency measurements. Broadband ultrasonic contact transducers with different center frequencies (1.0 MHz, 2.25 MHz, 5.0 MHz, and 10.0 MHz) were prepared and the micromechanical resonator was placed on the transducer, to which a 5-cycle tone burst signal was transmitted using a radio frequency ultrasonic pulser. The repetition rate was adjusted to 200 Hz to allow sufficient time for the vibration of the micromechanical resonator to fully decay before the next signal arrived.

The vibration was measured using a Michelson interferometer with a light source of a diode-pumped solid state laser (continuous wave at a 532 nm wavelength) in a single longitudinal mode. It was path-stabilized using an analog proportional-differential-integral controller and a piezo-actuated mirror to compensate the optical path length difference caused by low-frequency disturbances and noises. Intensity changes of fringe patterns were converted to voltage signals in a 50 MHz silicon fixed gain photodetector and recorded in a 500 MHz digital oscilloscope. The experiment was performed on an optic table with active self-leveling vibration isolation supports. Figure 4b shows a picture of the actual interferometer setup.

## 3. Theory and Calculation

**Figure 5:**
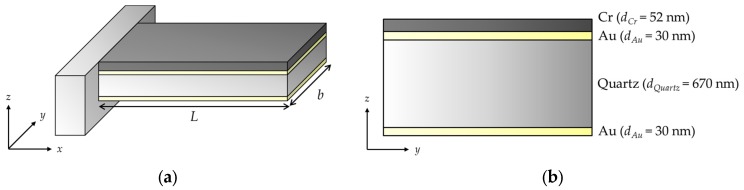
Dimensions of the micromechanical resonator: (**a**) 3D-view, *L* is the length and *b* is the width; (**b**) a cross-section, *d* is the thickness.

The resonant frequencies obtained from the experiment and corresponding vibration modes were verified using the Euler–Bernoulli beam theory [13]. Suppose that the chromium-coated micromechanical resonator is placed in the coordinate system shown in Figure 5. Again, the length and width of the structure are *L* and *b*, respectively.

The equation of motion of the beam is expressed in the form of a fourth order partial differential equation as follows:

(2) $$EI\frac{\partial^{4}w(x,t)}{\partial x^{4}}+\mu \frac{\partial^{2}w(x,t)}{\partial t^{2}}=0$$

where *E* is the Young’s modulus, *I* is the moment of inertia, *w*(*x*,*t*) is the deflection of beam as a function of length (*x*) and time (*t*), and *μ* is the mass per unit length. The term *EI* is also known as flexural rigidity. The resonant frequency for the *n*^th^ vibration mode, *f_n_*, can be obtained by solving the equation using the method of separation of variables. Suppose that the solution is expressed by the product of a spatial function, *X*(*x*), and a time function, *T*(*t*). Then, the deflection of beam is now written as follows:

(3) w(x,t)=X(x)T(t)

Then, Equation (2) becomes:

(4) $$\frac{EI}{\mu }\frac{1}{X}\frac{\partial^{4}X}{\partial x^{4}}=-\frac{1}{T}\frac{\partial^{2}T}{\partial t^{2}}$$

To satisfy Equation (4), it has to be equal to a constant, $w_{n}^{2}$, which is the square product of an angular frequency of the micromechanical resonator. Then, the left-hand side of Equation (4) is given as follows:

(5) $$\frac{\partial^{4}X}{\partial x^{4}}-k_{n}^{4}X=0$$

where $k_{n}^{4}$ is expressed as:

(6) $$k_{n}^{4}=\frac{\omega_{n}^{2}\mu }{EI}$$

From Equation (6), the resonant frequency of the micromechanical resonator is obtained as follows:

(7) $$f_{n}=\frac{1}{2\pi }\frac{{\left(k_{n}L\right)}^{2}}{L^{2}}\sqrt{\frac{EI}{\mu }}$$

where *k_n_L* is the coefficient associated with the vibration mode, and for a clamped-free boundary condition, *k*_1_*L* = 1.8751, *k*_2_*L* = 4.6941, and so on [13].

Since the micromechanical resonator is composed of four different layers, *EI* in Equation (7) is equal to the summation of the flexural rigidity of each layer:

*EI* = *EI_Au,bottom_* + *EI_Quartz_* + *EI_Au,top_* + *EI_Cr_* (8)

The moment of inertia for each layer is calculated using the parallel axis theorem as follows [14]:

(9) $$I_{Au,bottom}=\frac{1}{12}bd_{Au}^{3}+bd_{Au}{\left(h_{n}-\frac{d_{Au}}{2}\right)}^{2}$$

(10) $$I_{Quartz}=\frac{1}{12}bd_{Quartz}^{3}+bd_{Quartz}{\left(d_{Au}+\frac{d_{Quartz}}{2}-h_{n}\right)}^{2}$$

(11) $$I_{Au,top}=\frac{1}{12}bd_{Au}^{3}+bd_{Au}{\left(1.5d_{Au}+d_{Quartz}-h_{n}\right)}^{2}$$

(12) $$I_{Cr}=\frac{1}{12}bd_{Cr}^{3}+bd_{Cr}{\left(2d_{Au}+d_{Quartz}+\frac{d_{Cr}}{2}-h_{n}\right)}^{2}$$

where *d* is the film thickness. Additionally, *h_n_* is the neutral axis calculated as follows:

(13) $$\begin{array}{ll} h_{n} & =[E_{Au}d_{Au}^{2}/2+E_{Quartz}d_{Quartz}(d_{Au}+d_{Quartz}/2)+E_{Au}d_{Au}(1.5d_{Au}+d_{Quartz})\\ & +E_{Cr}d_{Cr}(2d_{Au}+d_{Quartz}+d_{Cr}/2)]/\left[2\left(E_{Au}d_{Au}\right)+E_{Quartz}d_{Quartz}+E_{Cr}d_{Cr}\right] \end{array}$$

Lastly, *μ* in Equation (7) is also equal to the summation of the mass per unit length of each layer:

(14) $$\mu =b(\rho_{quartz}d_{quartz}+2\rho_{Au}d_{Au}+\rho_{Cr}d_{Cr})$$

## 4. Results and Discussion

**Figure 6:**
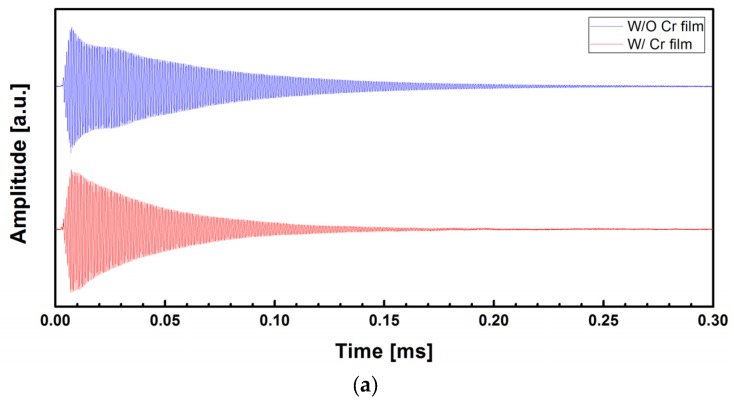

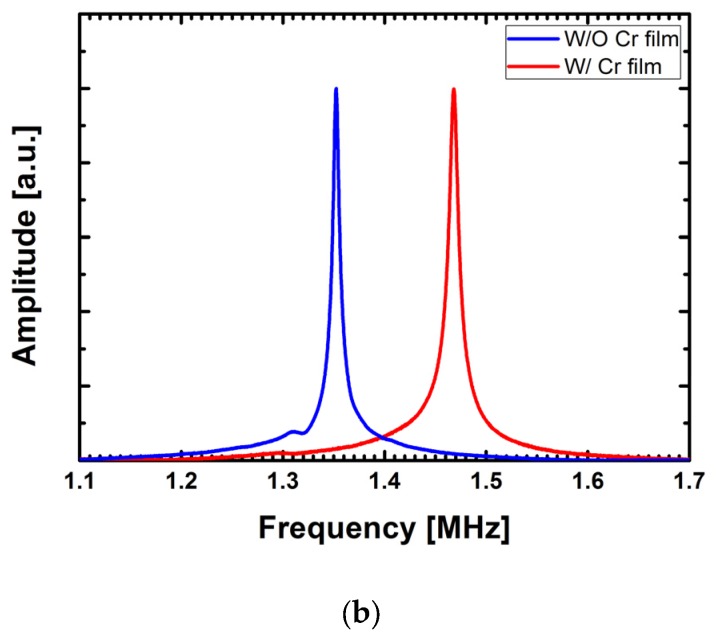
(**a**) Time-domain waveforms of the micromechanical resonators in the first flexural vibration mode; (**b**) frequency responses obtained from the fast Fourier transform (FFT) of the time-domain waveforms.

**Figure 7:**
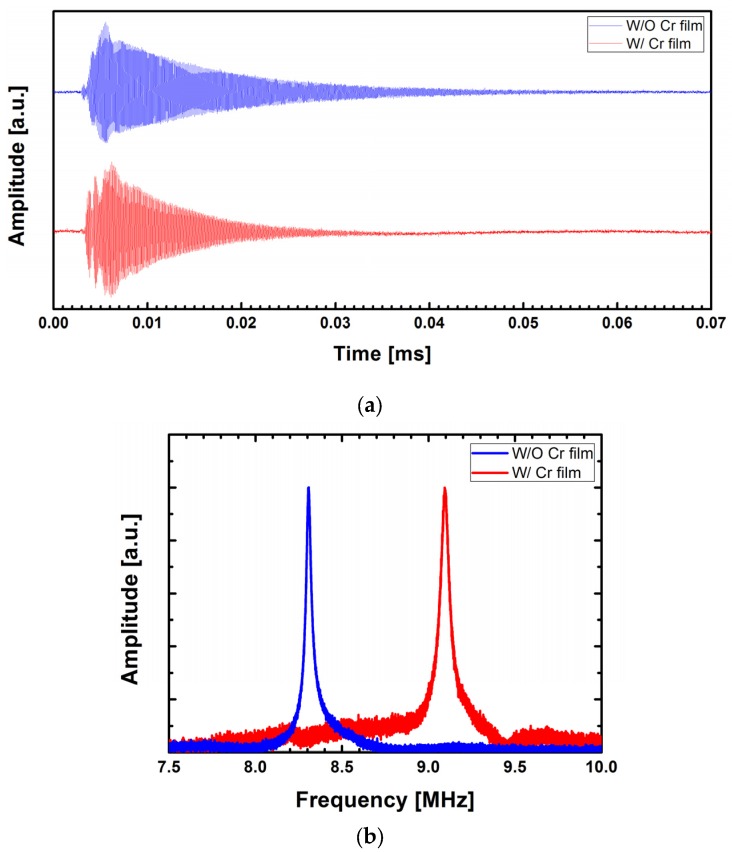
(**a**) Time-domain waveforms of the micromechanical resonators in the second flexural vibration mode; (**b**) frequency responses obtained from the fast Fourier transform (FFT) of the time-domain waveforms.

Figure 6 shows the measurement data for the 1st flexural vibration mode. The fast Fourier transform (FFT) of the time domain waveforms in Figure 6a was taken and the frequency responses are presented in Figure 6b. Resonant frequencies of 1.352 MHz and 1.468 MHz were obtained for the uncoated and Cr-coated micromechanical resonators, respectively. Similarly, the waveforms for the 2nd mode were presented in Figure 7. In this case, the resonant frequencies of 8.306 MHz and 9.093 were observed for the uncoated and Cr-coated micromechanical resonators, respectively.

**Figure 8:**
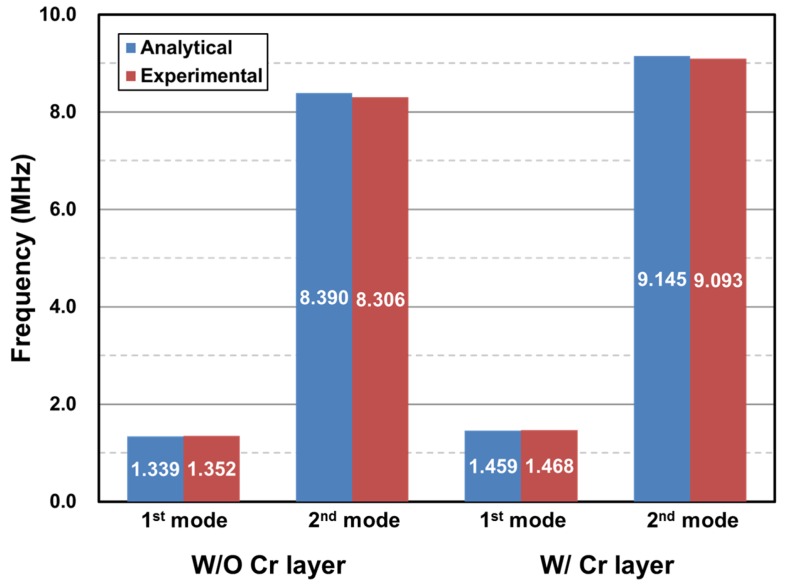
A comparison of resonant frequencies obtained from the experiment and the theory.

Using Equation (7) with material properties introduced in Table 1, *f*_1_ = 1.339 MHz and *f*_2_ = 8.390 MHz were obtained for the uncoated micromechanical resonator and taking the Young’s modulus of Cr film as a fitting parameter, *f*_1_ = 1.459 MHz and *f*_2_ = 9.145 MHz were obtained for *E_Cr_* = 139 ± 3 GPa, considering the local fluctuation of the film thickness. Meanwhile, the bulk properties of gold were used in the analytical estimation, even though the gold film is very thin (~30 nm). This was due to the fact that the scale effect on gold is not consistent, as summarized in Table 2. Although the elastic modulus of a gold thin-film often decreases with reduction of its thickness [15], studies show that the property is still comparable to the bulk value [16,17,18]. In addition, no dependency of the modulus on the film thickness was recognized in certain studies [19,20]. The scale effect on the gold films used in the present experiment is not yet clear, but the analytical estimations and experimental results agree excellently with each other, within 1% of the relative error as shown in Figure 8, implying that the scale effect may not be significant in this particular sample.

The elastic property of Cr in the present study is 49.8% lower than that of its bulk form, which is typically 248–279 GPa [21]. This scale effect is often observed in sub-micron Cr films and coatings for which Young’s modulus varies from 43 to 185 GPa [21,22,23].

The quality factor (*Q*) is an indication of damping characteristics in a resonating mechanical structure, and it is related to the performance aspects of the microresonator such as sensitivity and resolution. In general, a higher quality factor results in higher mass sensitivity, which is preferred for sensing, since the minimum detectable mass (Δ*m*_min_) is inversely proportional to *Q* [24]:

(15) $$\Delta m_{\min}\propto m/Q$$

where *m* is the mass. The quality factor in the *n*th vibrational mode (*Q_n_*) is determined as follows:

(16) $$Q_{n}=f_{n}/\Delta f_{n}$$

where Δ*f_n_* is the full width at half maximum of the resonance peak. From Figure 6b and Figure 7b, the values *Q*_1*,uncoated*_ = 148.5, *Q*_1*,Cr-coated*_ = 108.0, *Q*_2*,uncoated*_ = 207.5, and *Q*_2*,Cr-coated*_ = 113.6 were obtained. These values are higher than those of a 500 × 100 × 1 μm^3^ silicon microcantilever in air (*Q*_1_ = 19 and *Q*_2_ = 73) [25] and comparable to those of a 225 × 30 × 3 μm^3^ one (*Q*_1_ = 146.2) [7]. In addition, an increase of *Q* was observed with the higher vibration mode and this is consistent with what was reported [7,25].

The mass sensitivity (*S*) is again given by [7]:

(17) S=Δf/Δm

Since the Cr film has a volume of 20 × 10 × 0.052 μm^3^, *S* is calculated to be 1.6 Hz/fg for the first mode and 10.5 Hz/fg for the second mode. These are three orders of magnitude higher than those of conventional low-frequency silicon resonators [7,8,26], and thus the present study proposes a characterization technique with high sensitivity that makes it suitable for evaluating nanoscale thin-film materials.

## 5. Conclusions

The Young’s modulus of a 52 nm thick Cr film was evaluated by advanced micromechanical resonator testing. Actuation and detection of a micromechanical resonator oscillating in the high frequency range (~9.093 MHz) were performed using an ultrasonic tone burst signal generator and laser interferometry. The resonant frequencies for the first and second flexural vibration modes were analyzed using the Euler–Bernoulli beam theory and results showed that the measurement data are in excellent agreement with the theory, within 1% relative error. The resonant frequency shift upon deposition of the Cr layer revealed that the film’s elastic modulus is 139 ± 3 GPa, which is 49.8% smaller than its bulk value; this is attributable to the scale effect. This approach therefore provides an improved characterization technique for the Young’s modulus of nanoscale thin-films, with high accuracy and sensitivity up to 10.5 Hz/fg.

## Figures and Tables

**Table 1:** Material properties used in the analytical estimation.

Property	Material		
	Gold	Quartz	Chromium
Thickness (*d*) (nm)	30	670	52
Density (*ρ*) (kg/m^3^)	19,300	2200	7190
Young’s modulus (*E*) (GPa)	79	73	-

**Table 2:** Young’s moduli of gold thin-films from literature.

Reference	Fabrication Method	Characterization Method	Film Thickness (nm)	Young’s Modulus (GPa)
[15]	E-beam evaporation	Nanoindentation testing	100	55.5
			300	64.1
			500	88.76
[16]	Metal plasma immersion ion implantation and deposition	Microcantilever beam testing	19–62	69.1
[17]	E-beam evaporation	Microbeam testing	1000	57
		Nanoindentation testing		74
[18]	Multi-user microelectromechanical systems processes	Microcantilever beam testing	500	78
[19]	Sputtering	Microtensile testing	180	61.0
			310	49.5
			500	53.9
			680	53.1
			950	51.2
			1000	57.5
[20]	E-beam evaporation	Membrane deflection experiment	300	53–55
			500	
			1000	
